# Investigating predictors of community integration in individuals after stroke in a residential setting: A longitutinal study

**DOI:** 10.1371/journal.pone.0233015

**Published:** 2020-05-18

**Authors:** Isabela Matos, Adriana Fernandes, Iara Maso, Jamary Oliveira-Filho, Pedro Antônio de Jesus, Helena Fraga-Maia, Elen Beatriz Pinto

**Affiliations:** 1 Motor Behavior and Neurorehabilitation Research Group, Bahiana School of Medicine and Public Health, Salvador, Brazil; 2 Roberto Santos General Hospital, Salvador, Brazil; 3 Federal University of Bahia, Bahia, Brazil; 4 Bahia State University, Bahia, Brazil; University of Tasmania, AUSTRALIA

## Abstract

**Aim:**

To identify potential predictors of community integration in individuals after stroke using a residential setting-based strategy.

**Method:**

A prospective cohort of post-stroke individuals was recruited from the Stroke Unit of the Roberto Santos General Hospital (UAVC-HGRS). All included individuals were aged over 18 years, received a diagnosis of ischemic stroke confirmed by neuroimaging and resided in the city of Salvador (Bahia, Brazil). Following discharge from the stroke unit, the individuals themselves, or their responsible parties, were contacted by telephone to schedule a home visit no less than three months after discharge. All subjects were examined in their homes, at which time the Community Integration Questionnaire (CIQ) was also applied. A robust linear regression model was used to assess community reintegration using CIQ score as the outcome variable.

**Results:**

A total of 124 individuals effectively fulfilled the eligibility criteria: 51.6% were females, the median (IQR) age was 63(53–69) years, 82.3% were non-white, 53.2% were married, the median (IQR) of years of schooling was 6 (4–12) and family income averaged two minimum monthly wages. Investigated individuals presented a median (IQR) NIH Stroke Scale (NIHSS) score of 7 (4–12). Multivariate linear regression identified the following independent predictors of community integration: age (β = -0.095; 95% CI = -0.165 to -0.025; p = 0.008), diabetes mellitus (β = -2.348; 95% CI = -4.125 to -0.571; p = 0.010), smoking habit (β = -2.951; 95% CI = -5.081 to -0.821; p = 0.007), functional capacity upon hospital discharge (β = 0.168; 95% CI = 0.093 to 0.242; p = <0.001) and stroke severity (β = -0.163; CI = -0.318 to -0.009); p = 0.038).

**Conclusions:**

Regardless of length of time since stroke, individuals present restrictions that compromise their reintegration into their respective communities. The demographic, clinical and functional factors identified herein as potential predictors should be considered when conducting regular follow-up, as well as in the rehabilitation of individuals after stroke with the purpose to identify the interventions necessary to optimize their reintegration into the community.

## Introduction

Stroke impacts individuals not only due to its lethality, but also with respect to negative consequences in terms of changes in functional performance and the social life of survivors [1]. Despite advances in knowledge regarding the epidemiology, etiology, risk factors and treatment of stroke, information about life after this event remains limited [2,3], as well as in relation to environmental factors and the social participation of stroke victims living in the community [3].

The persistent side effects caused by stroke lead to relevant limitations in activities, generating a reduced perception of well-being in affected individuals [2,4]. A significant proportion of survivors persist with moderate or severe disability [5,6], which interferes in their return to activities and previous lifestyles, thereby hindering the social integration of these individuals within their communities [2,7]. Studies indicate that affected individuals suffer social isolation, restricted social participation and difficulties in reintegration [8,9]. Moreover, some acute and chronic predictors of reintegration success after stroke, such as advanced age, stroke severity, number of comorbidities, motor impairment and depression, have been found to be negatively associated with reintegration into the community [9,10,11], while a support network was positively associated with successful integration [10].

Social integration was previously identified as a predictor of life satisfaction after stroke [12]. However, little is known regarding the degree of reintegration into the community, especially in Brazil, making it necessary to expand our knowledge base surrounding aspects that interfere in the reintegration of these individuals within their communities. Considering the long-term needs of the survivors, diverse measures have been used to assess the result of reintegration, reflecting the complexity of this construct [13]. In general, researchers in this area have used subjective reports from patients or adapted questionnaires developed for specific scenarios; in some cases, no specific tools are employed to assess reintegration into the community [7,14].

The Community Integration Questionnaire (CIQ), a simple, reliable tool for assessing levels of integration at home and in the community, including the individual's perception and objective indicators [15], has not yet been used to assess community reintegration in stroke survivors in Brazil. Thus, the aim of this study was to evaluate community integration in individuals after stroke in a residential setting, as well as to identify predictors that could affect the successful reintegration.

## Methodology

### Study design and population

The present study employed a prospective cohort developed as part of a previous study [16]. Post-stroke individuals were recruited from the Stroke Unit of the Roberto Santos General Hospital (UAVC-HGRS). All included individuals were aged over 18 years, had received a diagnosis of ischemic stroke confirmed by neuroimaging (computed tomography or magnetic resonance imaging) and resided in the city of Salvador (Bahia, Brazil). Following discharge from the stroke unit, the individuals themselves, or their responsible parties, were contacted by telephone to schedule a home visit at least three months after discharge. Deceased individuals and those who had a new episode of stroke were excluded.

### Data collection procedures

Baseline data was collected from patient records contained in the previous study [16]. The scheduling of household visits was organized according to city districts to optimize resources. Interviews scheduled by telephone were generally conducted weekly from September 2016 and May 2018 when researchers, who were also neurorehabilitation specialists, were deployed to visit the post-stroke individuals’ homes. A sample size of 124 individuals was obtained considering a 95% confidence level, 80% power and, to detect differences significant changes in the degree of integration in the community, a difference of 2.2 points between mean values obtained using the Community Integration Scale (CIQ). All calculations were performed using software freely available at http://www.openepi.com/Menu/OE_Menu.htm.

### Variable definitions

The cohort database [16] was used to collect the following sociodemographic data: age, sex, schooling (years of study), self-reported skin color (for purposes of analysis, white or non-white), marital status (married or unmarried), family income (recorded in multiples of Brazilian minimum monthly wage), smoking status and alcohol consumption.

The following comorbidities were considered in patient medical records: systemic arterial hypertension (HTN), diabetes mellitus (DM), hypercholesterolemia, atrial fibrillation (AF) and other heart diseases.

Stroke severity was measured using the National Institutes of Health Stroke Scale (NIHSS) [17] during hospitalization at the stroke unit, with higher scores indicating greater severity (range: 0–42). The vascular territory of the lesion (anterior or posterior circulation), previous occurrence of stroke, thrombolytic treatment and hemorrhagic transformation were also recorded.

Functional capacity was assessed at the time of discharge using the Modified Barthel Index (MBI) [17]. Patients identified as aphasic were evaluated by speech therapists to functionally diagnose aphasia by clinical assessments of language, including spontaneous speech (directed and non-directed), listening and written comprehension, repetition, reading and writing [18].

The time elapsed between the stroke event and in-home interview was recorded in months. The Community Integration Questionnaire (CIQ) [19] was applied to assess the degree of integration each individual in their home and in their community. This questionnaire, which is divided into three domains: home integration, social integration and productive activity, consists of 15 questions. Total scores vary between 0 and 29, with higher scores indicating greater independence and integration.

In addition, information related to each individual’s surrounding physical environment was also collected, such as the presence of narrow sidewalks, obstacles on sidewalks, sidewalk irregularity, pedestrian crossings on nearby streets, and insufficient duration of crosswalk signals.

### Authorizations

This study received approved from the local institutional review board (protocol CAAE: 26412814.3.0000.5544) and all included participants provided a signed informed consent.

### Data analysis

Data analysis was conducted using Software for Statistics and Data Science (STATA V.14). Descriptive analysis employed means and standard deviations or median and interquartile range for numerical variables, while absolute and relative frequencies were considered for categorical variables. Since the outcome variable, integration into the community, presented non-normal distribution, a robust linear regression model was used. Multivariate analysis was conducted based on a theoretical model defined a priori to allow the operationalization of several explanatory variables. Associated factors were discriminated in hierarchical blocks, respecting the existing hierarchy between the levels of determination of integration in the community [20]. The hierarchy assumed that the previous block directly or indirectly affects subsequent ones. A forward strategy (anterograde process) was used to enter the blocks of variables in steps: the first block consisted of demographic and socioeconomic variables (distal determinacy); the second block included variables related to lifestyle and previous clinical conditions; the third block were related to stroke; the fourth block consisted of environmental aspects. Variables with demonstrated levels of statistical significance (p-value <0.20) remained in the adjusted model. The magnitude of association was estimated using a regression coefficient calculation, adopting a 95% confidence interval (95% CI) to determine predictive variables.

## Results

The baseline sociodemographic and clinical characteristics of the 124 individuals are described in Table 1. The studied population consisted of mostly females (51.6%) with a median (IQR) age of 63 years (53 to 69), 82.3% reported skin color as non-white and 53.2% were married. The median (IQR) number of years of schooling was 6 (4 to 12) and family income was 2 (1 to 2) monthly minimum wages. The most frequently reported lifestyle habits were alcoholic beverage consumption (25.8%) and smoking habit (18.5%).

**Table 1:** Sociodemographic, clinical, functional and lifestyle characteristics of 124 individuals seen at the stroke unit (UAVC-HGRS) in Salvador, Bahia-Brazil.

Variables collected at the stroke unit (UAVC-HGRS)	
**Sociodemographic variables**	
Age, median (IQR)	63 (53–69)
Female sex, n (%)	64 (51.6)
Non-white skin color, n (%)	102 (82.3)
Schooling in years, median (IQR)	6 (4–12)
Family income (minimum wages), median (IQR)	2 (1–2)
Marital status (married), n (%)	66 (53.2)
**Lifestyle habits**	
Alcoholic beverage consumption, n (%)	32 (25.8)
Smoking habit, n (%)	23 (18.5)
**Clinical**	
Stroke severity (NIHSS), median (IQR)	7 (4–12)
Posterior circulation involvement, n (%)	21 (16.9)
Hemorrhagic transformation, n (%)	11 (8.9)
Received Thrombolytic treatment, n (%)	35 (28.2)
Prior history of stroke, n (%)	28 (22.6)
Diagnosed as aphasic, n (%)	40 (32.3)
Functional capacity upon discharge (MBI), median (IQR)	48 (41–50)
Time since stroke in months, median (IQR)	20 (16–25)
**Comorbidities**	
Systemic arterial hypertension, n (%)	96 (77.4)
Diabetes mellitus, n (%)	41 (33.1)
Obesity, n (%)	19 (15.3)
Hypercholesterolemia, n (%)	9 (7.3)
Atrial fibrillation, n (%)	9 (7.3)
Other heart diseases, n (%)	30 (24.2)
**Aspects related to the physical environment**	
Presence of narrow sidewalks, n (%)	68 (54.8)
Presence of obstacles on sidewalks, n (%)	76 (61,3)
Sidewalks irregularity, n (%)	87 (70.2)
There are no pedestrian crossings on nearby, n (%)	56 (45.2)
Insufficient duration of crosswalk signals, n (%)	66 (53.2)

QR = interquartile range/ NIHSS = National Institutes of Health Stroke Scale

Stroke severity as measured by the NIHSS revealed a median (IQR) of 7 (4 to 12), indicating moderate stroke severity. Posterior circulation involvement was present in 16.9%, 8.9% suffered hemorrhagic transformation, 28.2% received thrombolytic treatment and 22.6% had a prior history of stroke. A total of 32.3% of the population were diagnosed as aphasic. Median (IQR) functional capacity upon discharge as measured by MBI was 48 (41 to 50), i.e. slightly dependent in the performance of their basic daily activities. The most prevalent comorbidities were HTN (77.4%), DM (33.1%) and other cardiopathies (24.2%). The median (IQR) elapsed time since stroke event and in-home visit was 20 months (16 to 25).

The studied individuals were assessed at their respective places of residence at a median time of 20 months (±7) after the stroke event. The mean overall CIQ score obtained was 8.9 (4.2 SD), with respective mean scores of 2.4 (2.0 SD) in the home integration domain, 5.9 (1.9 SD) in social integration and 0.5 (1.2 SD) in the productive activity domain (Table 2).

**Table 2:** Distribution of community integration according to the CIQ domains of individuals interviewed in their homes after stroke, Salvador, Bahia, Brazil.

CIQ	Max	Means (SD)
**Home integration**	10	2.4±2.0
**Social integration**	14	5.9±1.9
**Productive activity**	5	0.5±1.2
**Total**	29	8.9±4.2

The first phase of robust regression analysis identified that age upon admission to the stroke unit was significantly associated with CIQ score (Table 3). Even after adjusting for sociodemographic variables, this association remained significant. In fact, each additional five years of age resulted in an average reduction of 0.095 in overall CIQ (p = 0.008). In addition, educational level, income, skin color and sex were not found to significantly influence CIQ score. The second phase of the regression analysis included the block of the prior clinical and lifestyle habit variables adjusted by age. Smoking and DM were found to be significantly associated with CIQ, even after adjusting potentially confounding variables. Smokers presented an average reduction of -2.951 in CIQ score compared to non-smokers (p = 0.007). Individuals with DM also presented a reduction of -2.348 in average CIQ score (p = 0.010). The third phase of analysis, adjusted for relevant sociodemographic and clinical variables, identified that MBI was significantly associated (p <0.001) with CIQ, i.e. each increase of one point on MBI resulted in an average increment of 0.168 in CIQ score. The severity of stroke as assessed by NIHSS was also significantly associated (p = 0.038) with CIQ, i.e. each point of greater stroke severity was associated with a reduction of -0.163 in an individual’s CIQ score. It should be noted that the time since stroke was not found to significantly affect the resulting CIQ score. The final phase of the regression analysis did not identify any environmental aspects as being significantly associated with CIQ score, even after adjusting for the variables selected in the previous phases.

**Table 3:** Robust regression analysis, hierarchized in blocks, of associations between CIQ scores of individuals residing in a community after a stroke and sociodemographic, clinical and environmental variables, Salvador, Bahia, Brazil.

	CIQ score			
Variables	Crude robust regression coefficient and 95% CI		Adjusted robust regression coefficient and 95% CI	p value
**Distal level: Sociodemographic variables**^a^				
**Sex (female)**	0.907 (-0.665–2.480)	0.854 (-0.874–2.584)		0.330
**Age upon admission (continuous)**	-0.100 (-0.167 –-0.034)	-0.095 (-0.165 –-0.025)		**0.008**
**Schooling in years (over 8 years)**	0.115 (-0.069–0.301)	0.031 (-0.167–0.231)		0.753
**Marital status (married)**	0.107 (-1.552–1.767)	-0.107 (-1.846–1.630)		0.902
**Income (below R$ 937.00)**	0.455 (-0.087–0.999)	0.536 (-0.037–1.110)		0.067
**White skin color (vs. non-white)**	0.751 (-1.428–2.931)	0.917 (-1.365–3.200)		0.428
**Intermediary I level: Prior clinical variables and those related to lifestyle habits**^b^				
**Alcoholic beverage consumption (Yes)**	0.814 (-1.122–2.751)	1.427 (-0.529–3.384)		0.151
**Smoking habit (Yes)**	-2.618 (-4.753 –-0.483)	-2.951 (-5.081 –-0.821)		**0.007**
**Arterial hypertension (Yes)**	-0.258 (-2.240–1.723)	1.247 (-0.872–3.368)		0.246
**Diabetes Mellitus (Yes)**	-2.497 (-4.198 –-0.795)	-2.348 (-4.125 –-0.571)		**0.010**
**Obesity (Yes)**	0.442 (-1.865–2.749)	0.420 (-1.932–2.773)		0.724
**Dyslipidemia (Yes)**	-0.646 (-3.822–2.529)	0.085 (-3.061–3. 232)		0.957
**Atrial fibrillation (Yes)**	-1.256 (-4.491–1.977)	-1.137 (-4.249–1.950)		0.471
**Other cardiopathy (Yes)**	1.859 (-0.083–3.802)	1.305 (-0.629–3.240)		0.184
**Intermediary II level: Clinical variables related to the stroke**^c^				
**Barthel index upon hospital discharge (continuous)**	0.229 (0.164–0.295)	0.168 (0.093–0.242)		**<0.001**
**Use of thrombolytics (Yes)**	1.173 (-0.681–3.029)	0.094 (-1.663–1.851)		0.916
**Aphasia (Yes)**	-1.549 (-3.238–0.229)	0.061 (-1.583–1.707)		0.941
**Hemorrhagic transformation (Yes)**	-1.991 (-4.984–1.002)	-1.284 (-3.648–1.078)		0.284
**Vascular territory (Posterior circulation)**	0.157 (-2.053–2.369)	-0.956 (-2.822–0.910)		0.312
**Stroke severity (NIHSS)**	-0.260 (-0.399 –-0.122)	-0.163 (-0.318 –-0.009)		**0.038**
**Time since stroke in months (continuous)**	0.072 (-0.048–0.192)	0.033 (-0.065–0.132)		0.504
**Proximal level: Variables related to environmental aspects**^d^				
**Narrow sidewalks (Yes)**	-0.180 (-1.849–1.487)	0.228 (-1.272–1.730)		0.763
**Presence of obstacles on sidewalks (Yes)**	-1.709 (-3.408 –-0.011)	-0.916 (-2.444–0.610)		0.237
**Irregular sidewalks (Yes)**	1.632 -(0.175–3.441)	0.882 (-0.786–2.550)		0.297
**There are no pedestrian crossings in the streets (Yes)**	0.135 (-1.530–1.801)	0.451 (-1.025–1.927)		0.546
**Insufficient crossing time at the traffic lights (Yes)**	0.379 (-1.292–2.052)	0.119 (-1.396–1.636)		0.876

^**a**^Adjusted by the variables of the block /

^**b**^Adjusted by the variables of the block, sex, age upon admission and income /

^**c**^Adjusted by the variables of the block, gender, age upon admission, income, smoking and diabetes /

^**d**^ Adjusted by the variables of the block, sex, age upon admission, income, smoking, diabetes, Barthel after discharge and period of hospitalization at the UAVC. Abbreviations: NIHSS, National Institutes of Health Stroke Scale

## Discussion

The present study found that community reintegration was significantly influenced by advanced age, smoking habit, diabetes mellitus, impaired functional capacity as measured by MBI and stroke severity as measured by NIHSS. The older individuals in the present cohort experienced lower levels of integration into the community compared to younger individuals. Similar findings were reported by studies carried out in South Africa and Australia [21,22]. Moreover, a longitudinal study of a population with similar inclusion criteria as those herein also identified a strong association between advanced age and restricted integration after stroke [11].

A prospective cohort study that analyzed prior functional and occupational characteristics upon return to work 18 months after stroke found that older age was linked to a lower probability of returning to work [23]. These authors also found that a younger age was significantly associated with returning to work within 18 months after stroke, considering that return to work is of fundamental importance, as it not only plays an important role in the mental and occupational health of affected individuals, but also represents an objective measure of recovery and of the level of integration of these individuals into the community [23].

Similar to our results, a multicentric study by Verberne et al., 2018, reported that, in addition to age, stroke severity and the performance of daily activities as assessed by MBI were significantly associated with less restricted social participation by stroke survivors [2]. Even considering recent advances in stroke treatment, it is known that a significant portion of survivors present moderate or serious incapacity [6,7]. Motor sequelae are a frequent complication among stroke survivors, impacting the capacity of these individuals to live independently. A longitudinal study by Tornbom et al. (2016) assessed the impact of functional capacity on social integration one year after stroke by applying a self-assessment questionnaire measuring activity and social participation. These authors found that only physical aspects demonstrated a strong association with the self-perception of integration in the first, sixth and twelfth months after stroke [24].

Individuals with greater stroke severity presented reduced CIQ scores. While this finding demonstrates that affected individuals experience reduced community integration, it was not possible to identify the specific deficiencies related to stroke that significantly impacted the ability of individuals to reintegrate into their respective communities. While the present study did not detect a significant association between CIQ score and aphasia, it is worth noting that 32% of the individuals here were diagnosed as having impaired language skills. It is therefore possible that the NIHSS scores obtained herein were influenced by the presence of aphasia. Indeed, other studies have demonstrated an association between aphasia and poor community integration [25,26].

Some studies in stroke populations have reported low income levels as related to unfavorable outcomes. In general, most of these investigations were focused on individuals with low levels of education who were unemployed, which characterizes low socioeconomic status [27,28,29]. A cohort followed in the United States consisting of 1,965 stroke survivors demonstrated that stroke patients with these socioeconomic characteristics before stroke presented lower functional performance after stroke [28]. An association between a more unfavorable functional outcome and low socioeconomic status was also reported in another study by Song & Pan et al, 2017 [29]. The present study did not identify income as associated with community integration, which may have been due to homogeneity in our sample, as all included individuals were seen at a single stroke unit in a public hospital in Brazil, which generally serves economically less-favored populations.

Smoking is considered a well-established risk factor for the occurrence of stroke. The present study found that a smoking habit negatively affected the life of survivors, as smokers presented a mean reduction of over three points in CIQ score compared to non-smokers. While this relationship was not investigated in other studies, a retrospective international multicentric study demonstrated that smokers present worse functional results compared to nonsmokers three months after ischemic stroke [30]. A previous study investigated multifaceted constructs of the behavior of smokers and found an association between personality traits and smoking habit in adults and elderly individuals [31]. These authors speculated that individuals who smoke may exhibit a lack of perseverance, may not care about the consequences of their actions and could have limited self-discipline despite being aware of the harmful effects of smoking [32]. It is important to note that behavioral aspects were not specifically investigated in the present study.

While diabetes mellitus is an independent risk factor for stroke, this comorbidity has not been investigated with respect to the outcome of community integration, despite being associated with worse functional outcomes [33–36]. Our results indicate that DM is an independent predictor of reduced community integration. A study involving post-ischemic stroke individuals identified DM as a predictor of negative outcomes and related this comorbidity to higher mortality or functional dependency, as well as risk of recurrent stroke within 3–6 months after the first episode [33]. A more recent cohort study, with a similar population, sample size and instruments as those used this study, assessed the impact of DM on short and long-term results, comparing diabetic and non-diabetic individuals at 12 and 18 months after ischemic stroke and identified that the frequency of death and functional dependency were higher among diabetic post-stroke individuals [34]. Increased stroke severity and greater functional impairment were also reported in individuals with hyperglycemia who did not have a prior history of DM [35]_._ Psychological aspects of individuals living with diabetes was investigated in a national representative sample from The Netherlands. These authors found that people with type D personality (individuals with simultaneous experience of high level of negative affectivity and social inhibition) tend to experience less social support, greater social inhibition and solitude, which compromises how they face the consequences of disease [36] To date, it is not abundantly clear which resources in the environment help or hinder the reintegration of stroke survivors into the community. The present study selected some factors in the physical environment related to the infrastructure surrounding residences, since limited accessibility may restrict an individual’s ability to interact in an urban environment [37]. This is an important factor to be considered, as a lack of accessibility can lead to difficulties in mobility, especially for individuals with some type of limitation [3,38]. Despite the lack of a statistically significant association, inadequacies were identified in relation to the surrounding environmental conditions of the studied individuals.

### Strengths and limitations

This is the first Brazilian study to investigate community reintegration in a cohort of stroke survivors. The strength of this study lies in the fact that all assessments were made in the context of home visitation, i.e. in the real environment in which individuals interact. However, behavioral and cognitive aspects should be investigated as factors associated with community reintegration, despite the fact that these were not included in the present study.

## Conclusion

In conclusion, we found that, regardless of length of time since stroke, individuals present restrictions that compromise their reintegration into the community. The demographic, clinical and functional factors identified herein as potential predictors should be considered when conducting regular follow-up, as well as in the rehabilitation of individuals after stroke with the purpose to identify the interventions necessary to optimize their reintegration into the community. In Brazil, many individuals face difficulties in accessing primary care and insertion in rehabilitation programs, which represents a major challenge for current public policies.
